# Quality of life, diabetes-related stress and treatment satisfaction are correlated with glycemia risk index (GRI), time in range and hypoglycemia/hyperglycemia components in type 1 diabetes

**DOI:** 10.1007/s12020-024-03846-9

**Published:** 2024-05-24

**Authors:** Gonzalo Díaz-Soto, Paloma Pérez-López, Pablo Férnandez-Velasco, Pilar Bahillo-Curieses, María de la O Nieto de la Marca, Rebeca Jimenez, Daniel de Luis

**Affiliations:** 1 Endocrinology and Nutrition Department, Hospital Clínico Universitario Valladolid, Avenida Ramón y Cajal 3, Valladolid, CP: 47005 Spain; 2 Centro de Investigación en Endocrinología y Nutrición Clínica (CIENC), Universidad de Valladolid, Avenida Ramón y Cajal 3, Valladolid, CP: 47005 Spain; 3 Pediatrics Department, Hospital Clínico Universitario Valladolid, Avenida Ramón y Cajal 3, Valladolid, CP: 47005 Spain

**Keywords:** GRI, Quality of life, Diabetes related stress, TIR

## Abstract

**Introduction:**

To evaluate the relationship between the GRI -component of hypoglycemia (CHypo) and hyperglycemia (CHyper)- with diabetes quality of life (DQoL), diabetes-related stress (DDS), perception of hypoglycemia (Clarke Test), visual analogic scale (VAS) and diabetes-knowledge (DKQ2) in T1D.

**Methods:**

Cross-sectional study in 92 patients with T1D under intensive insulin treatment (21.7% CSII) and flash glucose monitoring (isCGM). Clinical, metabolic and glycometric parameters and quality of life/satisfaction questionnaires were analyzed.

**Results:**

92 patients (54.3% male, BMI 25.4 ± 4.5 kg/m^2^, HbA1c 7.5 ± 1.0%, TIR 53.9 ± 15.9%) with mean age 36.1 ± 12.6years and 17.8 ± 11.3 T1D duration. The mean GRI was 60.6 ± 22.2 with a CHypo and CHyper of 5.9 ± 4.8 and 27.3 ± 14.4, respectively. 19.1% presented a pathological Clarke’s test. Patients with TIR > 70% and GRI < 40 showed better VAS (8.8 ± 1.3 vs 9.3 ± 0.9, *p* < 0.05) and DDS (46.4 ± 22.1 vs 36.7 ± 16.6, *p* < 0.05) scores, showing no differences between groups. CHyper > 15 and Chypo > 3.4 were related to worse levels of DQoL (91.1 ± 23.9 vs 76.6 ± 18.6 and 94.6 ± 24.8 vs 79.8 ± 20.1, *p* < 0.01), DDS(49.8 ± 22.4 vs 35.7 ± 16.5 and 49.8 ± 22.4 vs 35.7 ± 16.5, *p* < 0.01),and DKQ2 (24.4 ± 4.3 vs 26.8 ± 5.2 and 24.1 ± 4.8 vs 26.0 ± 4.6, *p* < 0.05), respectively. Worse metabolic control defined by GRI correlated with worse scores in VAS (*r* = −0.209, *p* < 0.05), DQoL (*r* = 0.205, *p* < 0.05), and DDS (*r* = 0.205, *p* < 0.05). No difference was observed in knowledge´s scale. CHyper correlated with worse scores in VAS (*r* = −0.231, *p* < 0.05), DQoL (*r* = 0.422, *p* < 0.01), and DDS (*r* = 0.341, *p* < 0.01) and lower degree of knowledge DKQ2 (*r* = −0.231, *p* < 0.05). When analyzing DQoL as a dependent variable in a multiple lineal regression, only age (β = 0.747; *p* < 0.001) and CHyper (β = 0.717; *p* < 0.001) maintained statistical significance.

**Conclusions:**

Higher GRI was related to worse quality of life, diabetes-related stress and satisfaction with treatment, analogous to the TIR results.CHyper an Chypo were related to a greater decline in quality of life, diabetes-related stress, and lower satisfaction with treatment.However, in a multiple linear regression, only CHyper maintained statistical significance.

## Introduction

Data provided by continuous glucose monitoring (CGM) systems have become the preferred form of blood glucose monitoring to achieve adequate control in patients with type 1 diabetes (T1D) [1–3]. It is well known that the use of these devices improves glycosylated hemoglobin A1c (HbA1c) and Time in Range (TIR) levels, reducing the number of episodes of acute hypoglycemia and chronic hyperglycemia and glycemic variability, including acute decompensation and hospital admissions [4–7]. It has also been related to a decrease in the risk of long-term complications and improvement in quality of life of T1D [4, 8–10].

The quality of life of patients with T1D involves different factors of the psychosocial sphere such as stress related to the disease, the grade of knowledge or satisfaction with the treatment, among others [11]. The variability of the scales used to evaluate them, together with the fact that not all of them are examined in the different studies, makes it difficult to compare them [9–13]. However, this is an important point to bear in mind, since improvements in the quality of life of T1D are associated with improvements in parameters such as the TIR, and vice versa [10].

Recently, the appearance of the Glycemic Risk Index (GRI) [14] as a new parameter to measure the quality of glycemic control in patients with diabetes has demonstrated its usefulness in adult and pediatric patients in clinical practice [15, 16]. In addition, GRI has proved its simplicity, ease of calculation and interpretation and its good correlation with the other parameters of the CGM, especially with TIR [17, 18]. The GRI consists of two components, one for hypoglycemia (CHypo) and one for hyperglycemia (CHyper). Both components are calculated from the respective times below and above range weighted according to their clinical relevance. In addition, the GRI can be categorized and graphically represented by percentiles (Pc) in five zones, from the best (Pc: 0–20) to the worst (Pc: 80–100) glycemic control.

However, despite the advantages of integrating this new glycemic metric into the Ambulatory Glucose Profile (AGP) report, its recent appearance means that its relationship with the psychosocial sphere of adult patients with T1D has not yet been elucidated.

The aim of the present study was to evaluate the relationship between the GRI and its CHypo and CHyper, with T1D quality of life, diabetes-related stress, perception of hypoglycemia, satisfaction with treatment, and degree of knowledge in a cohort of adult patients with T1D.

## Material and methods

### Participants

Cross-sectional study in 92 adult patients with T1D with stable control (more than 1 year of diabetes diagnosis and more than 3 months without changes in insulin treatment) on intensive insulin treatment (multiple doses of insulin (MDI) or continuous subcutaneous insulin infusion (CSII)) and flash glucose monitoring (isCGM) (Free Style Libre 2, Abbott Diabetes Care, Witney, UK), under follow-up in a tertiary Hospital.

### Procedures

Clinical and glycometric parameters were collected from the isCGM system data download platform of all patients who attended a control visit between June and December 2021, including in the report the last 14 days prior to the visit [19]. Patients with insufficient use of the system (<70%) and those with recent changes in treatment regimen (insulin type or initiation of CSII) or with less than 3 months of isCGM use were excluded. HbA1c was also measured between 7 and 10 days before the patient’s visit by turbidimetric inhibition immunoassay standardized to the National Glycohemoglobin Standardization Program (Roche Diagnostics, Geneva, Switzerland). Glycometric data were defined as mean glucose (mg/dL), glucose management indicator (GMI) (%), coefficient of variation (CV) (%), and percentages of TIR, time above range (TAR) and time below range (TBR) (%). The latter were divided into Very low, when blood glucose was less than 54 mg/dL (<3.0 mmol/L) TBR < 54; Low, between 54 and 70 mg/dL (3.0–3.9 mmol/L) TBR 54–70; in Range, between 71 and 180 mg/dL (4.0–10.0 mmol/L); High, between 181 and 250 mg/dL (10.1–13.9 mmol/L) TAR 180–250; and Very high, above 250 mg/dL (>13.9 mmol/L) TAR > 250. From the data of the different times obtained from the isCGM, the Hypoglycemia and Hyperglycemia components of the GRI were calculated, whose formula is developed as follows: CHypo = (TBR < 54) + (0.8 × TBR 54–70). CHyper = (TAR > 250) + (0.5 × TAR 180–250). GRI = (3.0 × CHypo) + (1.6 × CHyper) [14]. The education level of the patients in the study (divided into primary, secondary and higher education levels) was collected.

The following validated questionnaires were evaluated at the time of the visit, (1) Diabetes mellitus-specific quality of life questionnaire 20 in Spanish version (DQoL) [11]. The DQoL assesses 4 spheres (a) satisfaction (b) impact (c) social/vocational concern and (d) diabetes-related concern. A lower score indicates better quality of life: range 43–215. (2) The Spanish version of the diabetes-related distress scale (DDS) [20]. The DDS assess the level of diabetes-related patient stress in four subscales: emotional burden, physician-related distress, treatment-related distress, and interpersonal distress. The score range is 17 to 102, with the higher score being related to the greater degree of stress. (3) Clarke questionnaire for the perception of hypoglycemia, Spanish version [21] (1–2R normal perception; 3R indeterminate perception; >3R hypoglycemia unnoticed). The diabetes knowledge questionnaire was evaluated in its Spanish version (DKQ2) [22], which estimates the degree of knowledge, the maximum degree being a score of 26. Finally, a global assessment of quality of life was made using the Visual Analog Scale (VAS) from 0 to 10 points, with the maximum quality of life being 10 points.

### Statistical analysis

Quantitative variables were expressed as mean and (standard deviation) if normally distributed or as median and [interquartile range] when the distribution was not normal. Qualitative variables were expressed in terms of percentages. Comparison between qualitative variables was performed by the Chi-square test, using Fisher’s exact test where necessary. The normal distribution of quantitative variables was examined using the one-sample Kolmogorov–Smirnov test. The quantitative variables with normal distribution were analyzed using a bilateral Student’s *t*-test, and non-parametric variables were evaluated by using the Mann–Whitney U test. The association of quantitative variables was calculated using Pearson’s linear correlation coefficient. A stratified analysis of the main variables was performed by level of GRI > 40 (poor glycemic control) or ≤40 (group with better glycemic control), CHypo greater or less than 3.4, CHyper greater or less than 15 and TIR greater or less than 70%, as previously described [10]. Finally, a multiple lineal regression model was used that incorporated as independent variables the effect of CHypo, CHyper, type of treatment, CV, age, sex, years of duration of diabetes and education level on DQoL as dependent variable. For all calculations, a p-probability of less than 0.05 was considered significant. SPSS 23.0 (SPSS Inc., Chicago, IL, USA) and Rstudio version RStudio Team (2022 PBC, Boston, MA) were used for data analysis.

All patients signed an informed consent for their inclusion before participating in the study. The protocol was approved by the Clinical Research Ethics Committee of our Institution, and the study was conducted in accordance with the Declaration of Helsinki.

## Results

A total of 92 patients with T1D (54.3% male) were evaluated, with a mean age of 36.1 (12.6) years, 17.8 (11.3) years of T1D evolution and a mean BMI of 25.4 (4.5) kg/m^2^. The mean HbA1c was 7.5 (1.0)%. 21.7% were under CSII. The mean number of daily scans was 9.8 (5.4) with a mean percentage of device use of 91.2 (10.6)%. The glycometric measures obtained were: mean blood glucose 171.5 (35.8) mg/dl, mean SD 67.4 (20.8) mg/dl, CV 40.4 (7.3)%; TIR 53.9 (15.9)%, TBR < 4 2.4 [0.0–13.0]%; TBR 54–70 4.3 (2.8)%; TAR 180–250 24.1 (8.0)%; TAR > 250 15.2 (12.7)%. Education level of patients of the study was: Primary education: 14.1%, Secondary education: 56.5% and Higher education: 29.3% (Table 1). Differences between CSII and MDI are shown in Supplementary Files.

**Table 1:** Clinical, metabolic, glucometric and psychosocial features of patients with T1D

Parameter	Mean (standard deviation)
Number of patients	92
Gender (% male)	54.3
Mean age (years)	36.2 (12.6)
Duration of diabetes (years)	17.8 (11.4)
CSII (%)	21.7
Mean HbA1C (%)	7.5 (1.0)
NFCC mmol/mol	58 (10.0)
Mean glucose (mg/dl)	171.5 (35.8)
N° daily scans	9.8 (5.4)
% Sensor Use	91.2 (10.6)
% TIR (70–180 mg/dL)	53.9 (15.9)
% TAR (>250 mg/dL)	15.2 (12.7)
%TAR (181–250 mg/dL)	24.1 (8.0)
% TBR (54–69 mg/dL)	4.3 (2.8)
% TBR (54 mg/dL)	2.4 [0.0–13.0]
SD (mg/dl)	67.4 (20.8)
CV (%)	40.4 (7.3)
GMI (%)	7.6 (1.2)
GRI	60.6 (22.2)
GRI ZONE A % (P1-20)	5.4
GRI ZONE B % (P21-40)	10.9
GRI ZONE C % (P41-60)	34.8
GRI ZONE D % (P61-80)	30.4
GRI ZONE E % (P81-100)	18.5
CHypo	5.9 (4.8)
CHyper	27.3 (14.4)
DQoL TOTAL	85.8 (23.1)
DQoL satisfaction	32.0 (9.6)
DQoL impact	32.8 (9.5)
DQoL social concern	12.3 (5.5)
DQoL diabetes concern	9.7 (6.2)
DDS TOTAL	44.8 (21.5)
DDS emotional burden	14.1 (6.4)
DDS physician distress	9.1 (7.2)
DDS regimen distress	14.4 (7.0)
DDS interpersonal distress	6.9 (4.5)
DKQ2	25.2 (4.8)
VAS	8.9 (1.2)
Clarke > 3 (% positive)	19.1
Education level (%)	Primary education: 14.1
	Secondary education: 56.5
	Higher education: 29.3

*T1D* type 1 diabetes, *CSII* continuous subcutaneous insulin infusion, *MDI* multiple daily insulin injections, *TIR* time in range, *TAR* time above range, *TBR* time below range, *CV* coefficient of glycemic variability, *GMI* glucose management indicator, *SD* standard deviation, *GRI* glycemia risk index, *CHypo* hypoglycemia component, *CHyper* hyperglycemia component, *DQoL* diabetes quality of life, DQoL satisfaction category, *DQoL* impact category, *DQoL* social concern category, *DQoL* diabetes concern category, *DDS* diabetes distress scale, *DDS* emotional burden category, *DDS* physician distress category, *DDS* regimen distress category, *DDS* interpersonal distress category, *DKQ2* diabetes knowledge questionnaire 2, *VAS* Visual Analogic Scale, *Clarke* Clarke’s questionnaire, *NS* not significant

**Fig. 1:**
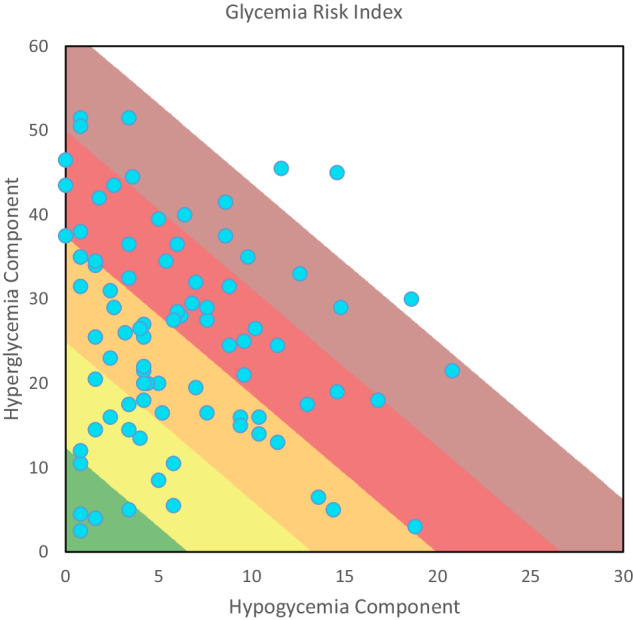
Glycemia risk index (GRI) grid showing the hyperglycemia component versus hypoglycemia component

The mean GRI was 60.6 (22.2) with a Hypo and Hyperglycemia Component of 5.9 (4.8) and 27.3 (14.4), respectively. As for the distribution of the GRI by zones, 5.4% occupied zone A (Pc: 0–20), 10.9% zone B (Pc: 21–40), 34.8% zone C (Pc: 41–60), 30.4% zone D (Pc: 61–80) and the remaining 18.5% zone E (Pc: 81–100). The mean scores on the questionnaires were: DQoL 85.8 (23.1), DDS 44.8 (21.5), VAS 8.9 (1.2) and DKQ2 25.2 (4.8). Clarke’s test showed hypoglycemia unawareness in 19.1% of the patients (Table 1 and Fig. 1).

Those patients with better glycemic control define with a TIR > 70% or GRI < 40 showed better scores in the scales: VAS (8.8 ± 1.3 vs 9.3 ± 0.9; *p* < 0.05) and DDS (46.4 ± 22.1 vs 36.7 ± 16.6; *p* < 0.05). No differences between TIR and GRI scale results was found. On the other hand, the group with CHyper > 15 (worse control) was related to worse results in the questionnaires: DQoL (91.1 ± 23.9 vs 76.6 ± 18.6; *p* < 0.01), DDS (49.8 ± 22.4 vs 35.7 ± 16.5; *p* < 0.01), and DKQ2 (24.4 ± 4.3 vs 26.8 ± 5.2; *p* < 0.05). Similarly, CHypo > 3.4 was associated to worse results in DQoL (94.6 ± 24.8 vs 79.8 ± 20.1; *p* < 0.01), DDS (49.8 ± 23.1 vs 41.4 ± 19.9; *p* < 0.01), and DKQ2 (24.1 ± 4.8 vs 26.0 ± 4.6; *p* < 0.05) (Table 2).

**Table 2:** Relationship between the scores on quality of life questionnaires and the values of GRI andvTIR

Parameters	GRI ≤ 40	GRI > 40	*p*-value	TIR ≥ 70%	TIR < 70%	*p-*value
DQoL	80.5 (22.4)	86.5 (23.3)	ns	78.1 (22.7)	87.3 (23.1)	ns
DDS	36.7 (16.6)	46.4 (22.1)	*P* < 0.05	36.2 (16.9)	46.5 (22.0)	*P* < 0.05
DKQ2	25.8 (5.4)	25.1 (4.7)	ns	25.9 (5.2)	25.1 (4.7)	ns
VAS	9.3 (0.9)	8.8 (1.3)	*P* < 0.05	9.3 (0.8)	8.8 (1.3)	*P* < 0.05

Parameters	CHyper ≤ 15	CHyper > 15	*p*-value	CHypo < 3.4	CHypo > 3.4	*p*-value
DQoL	76.6 (18.6)	91.1 (23.9)	*P* < 0.01	79.8 (20,1)	94.6 (24.8)	*P* < 0.01
DDS	35.7 (16.5)	49.8 (22.4)	*P* < 0.01	41.4 (19.9)	49.8 (23.1)	*P* < 0.05
DKQ2	26.8 (5.1)	24.4 (4.3)	*P* < 0.05	26.0 (4.6)	24.1 (4.8)	*P* < 0.05
VAS	9.3 (0.8)	8.8 (1.3)	ns	8.9 (1.1)	8.8 (1.4)	ns

*GRI* glycemia risk index, *CHypo* component of hypoglycemia, *CHyper* component of hyperglycemia, *TIR* time in range, *DQoL* diabetes quality of life, *DDS* diabetes distress scale, *DKQ2* diabetes knowledge questionnaire 2, *VAS* visual analogic scale

**Fig. 2:**
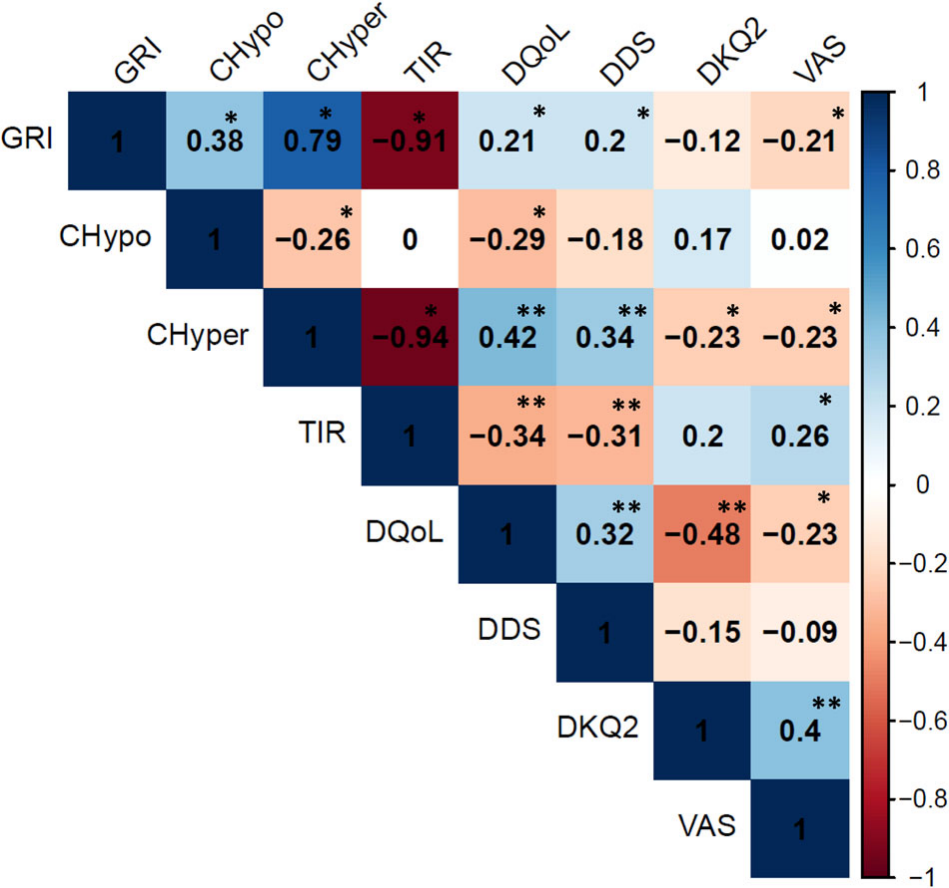
Correlation between glycometric parameters and psychosocial features. GRI glycemia risk index, CHypo component of hypoglycemia, CHyper component of hyperglycemia, TIR time in range, DQoL diabetes quality of life, DDS diabetes distress scale, DKQ2 diabetes knowledge questionnaire 2, VAS visual analogic scale, NS not significant, R Pearson’s correlation coefficient, *P < 0.05. **P < 0.01

Worse metabolic control defined by GRI correlated with worse scores in VAS (*r* = −0.209; *p* < 0.05), DQoL (*r* = 0.205; *p* < 0.05), and DDS (*r* = 0.205, *p* < 0.05); parallel to the group with higher TIR: VAS (*r* = 0.262; *p* < 0.05), DQoL (*r* = −0.344; *p* < 0.01), and DDS (*r* = −0.313; *p* < 0.01). No correlation was observed in DKQ2. CHyper was correlated with worse scores in: VAS (*r* = −0.231; *p* < 0.05), DQoL (*r* = 0.422; *p* < 0.01) and DDS (*r* = 0.341; *p* < 0.01), as well as with a lower degree of knowledge DKQ2 (*r* = −0.231; *p* < 0.05). CHypo was weakly correlated with DQoL (*r* = −0.294, *p* < 0.05) (Fig. 2).

No difference was shown in GRI or in the rest of the glycometrics with respect to the positive or negative Clarke test (Supplementary files). There were also no significant differences between questionnaires score in the percentile group (A-E) by GRI. However, there was a tendency towards greater satisfaction with treatment VAS (9.5 ± 0.9 group A vs 8.6 ± 0.8 group E; *p* = 0.449) and better quality of life DQoL (80.8 ± 26.3 group A vs 85.8 ± 23.2 group E; *p* = 0.302) in those with better GRI. The diabetes related-stress was higher in the most extreme percentiles DDS (44.2 ± 20.8 group A, 33.0 ± 13.7 group B, 45.6 ± 23.9 group C, 46.1 ± 19.3 group D, 48.5 ± 24.1 group E; *p* = 0.461), with no differences in DQK2 among the different groups (Table 3).

**Table 3:** Relationship between GRI zones and the scores on quality of life tests

Parameters	DQoL	DDS	DKQ2	VAS
GRI ZONE A	80.8 (26.3)	44.2 (20.8)	24.6 (5.9)	9.5 (0.9)
GRI ZONE B	80.4 (21.8)	33.0 (13.8)	26.4 (5.3)	9.2 (0.9)
GRI ZONE C	82.8 (20.5)	45.6 (23.9)	26.1 (5.2)	9.0 (1.5)
GRI ZONE D	85.4 (21.7)	46.1 (19.3)	24.2 (4.6)	8.6 (0.8)
GRI ZONE E	96.5 (28.8)	48.5 (24.1)	24.8 (3.4)	8.9 (1.2)
*p*-value	ns	ns	ns	ns

*GRI* glycemia risk index, *DQoL* diabetes quality of life, *DDS* diabetes distress scale, *DKQ2* diabetes knowledge questionnaire 2, *VSA* visual analogue scale

No differences were observed between the scores on the different questionnaires and educational level, with the exception of the degree of knowledge (DKQ2), where it was found that a higher education level was associated with a higher score on the questionnaire (primary 22; secondary 26.2, and higher education 26.2) (*p* < 0.05).

Finally, when analyzing DQoL as a dependent variable in a multiple lineal regression model that included CHypo, CHyper, type of treatment, CV, age, sex, years of duration of diabetes and education level as independent variables, the only variables that maintained statistical significance were age (β = 0.747; *p* < 0.001) and CHyper (β = 0.717; *p* < 0.001) (Table 4).

**Table 4:** Multiple linear regression model to predict quality of life

Predictors	B	*p*-value	95% CI
Gender	−8.203	ns	[−17.362 to 0.956]
Mean age	0.759	<0.01	[0.267–1.251]
Duration of diabetes	−0.313	ns	[−0.837 to 0.213]
Type of treatment	2.296	ns	[−8.770 to 13.362]
CHypo	−0.644	ns	[−2.108 to 0.821]
CHyper	0.714	<0.01	[0.347–1.080]
CV	0.069	ns	[−0.912 to 1.050]
Education level	0.530	ns	[−7.170 to 8.231]

*B* regression coefficient, *CI* confidence interval, *CHypo* hypoglycemia component, *CHyper* hyperglycemia component, *CV* coefficient of glycemic variability, *NS* not significant

## Discussion

The GRI as a new parameter of glycemic control has aroused great interest among professionals dedicated to diabetes care [14]. Despite its recent appearance, it has been shown to correlate with glycometric variables such as TIR, TBR, TAR or Coefficient of variation (CV) [17, 18]. In fact, recent studies have related TIR to long-term microvascular complications (retinopathy, nephropathy) [23, 24]. However, its relationship with different aspects of the psychosocial sphere has not been yet studied in adults.

Different authors have already demonstrated the relationship between the TIR and the quality of life of T1D [10, 12, 25]. However, this is to our knowledge the first article that relates the GRI and its two components (hypo and hyperglycemia) to different parameters of the psychosocial sphere in adults with T1D. Our results show how GRI is related to diabetes-related stress (DDS), quality of life (DQoL) and overall treatment satisfaction (VAS) in adult patients with T1D. However, and despite the weighting of the CHypo and CHyper according to their clinical significance [14], this relationship is similar to that obtained by TIR [17, 18]. In fact, the similar results obtained in those patients with better glycemic control (TIR > 70% and GRI < 40) demonstrate the lack of superiority of either of the two glycometrics when evaluating the psychosocial sphere of T1D adult.

So far, only one Italian group [10] has evaluated the satisfaction of an exclusively pediatric cohort and its relationship with GRI using a CGM satisfaction questionnaire (CGM-SAT). This cross-sectional study shows how satisfaction with CGM was significantly related to TIR and negatively related to GRI, in line with what was observed in our study. However, Marigliano’s study uses questionnaires specifically aimed at assessing satisfaction with a given CGM system in a pediatric population (with the difficulties inherent to the assessment of satisfaction in childhood). Moreover, it does not use specific scales to assess diabetes-specific quality of life, disease stress, global quality of life, degree of knowledge or perception of validated hypoglycemia.

Given the close correlation between TIR and GRI [17, 18], our results are partly to be expected. However, the weighting of CHypo in GRI calculation [14] due to its clinical importance could theoretically lead us to expect a better correlation of quality of life with GRI. Our study failed to demonstrate a superiority of the GRI when assessing the degree of quality of life, nor correlation with the degree of diabetes-related stress or quality of life with TBR or TAR. But it supports the individual evaluation of both components of Hyper and Hypoglycemia. In fact, CHyper and CHypo were more strongly associated with worse scores in DQoL, VAS, and DDS; and to a lower degree of knowledge (DKQ2). In the multiple linear regression model to predict DQoL, only CHyper and age maintained statistical significance, not CHypo, despite being the most penalized factor in the GRI calculation, and other variables such as education level showed no difference. It may be due to two factors: Although the patients evaluated were randomly selected and those with unawareness hypoglycemia were not excluded, the degree of overall hypoglycemia in our population is not high (TBR 54–70 4.3 (2.8)). In this sense, the T1D patients evaluated could have related their quality of life more to the CHyper than to the risk of hypoglycemia. Not surprisingly, the highest correlation among all those glycometric evaluated was between CHyper with DQoL (*r* = 0.422; *p* < 0.05). Furthermore, the fact that we found no differences in GRI and its components between patients with a positive or negative Clarke’s test supports the thesis of an underrepresentation of patients with undetected hypoglycemia, a subgroup that would penalize quality of life in a greater way in relation to CHypo. Nevertheless, our results are congruent with the Marigliano et al. study where no significant relationships were observed between satisfaction with CGM and glycometric such as TBR, TAR or CV [10]. Furthermore, the positive results in the psychosocial sphere in those subjects with better glycemic control by CHyper could be related to a greater awareness of the disease, as well as a greater use of therapeutic education programs, which would be reflected in a higher degree of knowledge in the group with CHyper < 15.

Furthermore, although no differences were observed between the GRI groups (A–E) and the different questionnaires, there does seem to be a tendency in those patients with better GRI (Groups A and B) towards greater satisfaction with treatment and better quality of life, with the stress level being higher in the more extreme groups (A, D and E). In group A, it could be related to a greater self-demand/burden related to the disease for optimal control of their diabetes.

Our study has certain limitations. First, it is a single-center cross-sectional study with a relatively small sample size compared to big data studies; however, it is a real-life cohort with stable control, with different types of treatment (MDI and CSII) and comprehensive knowledge of glycometric and clinical variables with a single current CGM system where quality of life, knowledge and degree of diabetes-related stress have been comprehensively assessed in a systematic and representative manner. Furthermore, our results are in line with the few studies published to date on quality of life and TIR [5, 12] and on the relationship between TIR and GRI [14, 17, 18]. Our study also has strengths, this the first to relate GRI and its components in T1D to quality of life, diabetes-related stress, perception of hypoglycemia, satisfaction with treatment, and degree of knowledge. In addition, it sheds light on new glycometric parameters that may be important in the future when assessing quality of life in subjects with diabetes. In this sense, it is important to mention that the weak correlation found between the different variables evaluated is not due to low statistical power but to the difficulty of finding significant relationships in a complex sphere as the psychosocial [12]. Further longitudinal evaluation studies and studies in specific populations (especially those at high risk of hypoglycemia or unawareness), as well as in other types of diabetes, are necessary to demonstrate the present results.

In conclusion, the GRI correlated with improved outcomes in quality of life, diabetes-related stress, and satisfaction with treatment, with no differences in the level of knowledge or perception of hypoglycemia, analogous to the TIR. The parameters that were related to a greater decline in quality of life, diabetes-related stress, and lower satisfaction with treatment were CHyper and Chypo. However, in a multiple linear regression, only CHyper maintained statistical significance.

## Supplementary information

Supplementary tables

## Data Availability

The data that support the findings of this study are not openly available due to reasons of sensitivity and are available from the corresponding author upon reasonable request. Data are located in controlled access data storage at Valladolid University.

## Abbreviations

GRI: glycemia risk index
CHypo: component of hypoglycemia
CHyper: component of hyperglycemia
DQoL: diabetes quality of life questionnaire
DDS: diabetes-related distress scale
VAS: visual analogic scale (VAS)
DKQ2: diabetes knowledge questionnaire
TIR: time in range (70–180 mg/dl)
TBR: time below Range (<70 mg/dl)
TAR: time above range (>180 mg/dL)
T1D Type: 1 diabetes
isCGM: flash glucose monitoring or intermittent scanned continuous glucose monitoring
CGM: continous glucose monitoring
AGP: ambulatory glucose profile
HbA1c: glycated hemoglobin
MDI: multiple doses of insulin
CSII: continuous subcutaneous insulin infusion
Pc: percentiles
GMI: glucose management indicator (GMI)
CV: coefficient of variation
TBR<54: time below 54 mg/dL
TBR 54–70: time between 54 and 70 mg/dL in Range
TAR 180–250: time between 81 and 250 mg/dL
TAR>250: time above 250 mg/dL
