# Supplementary material for: Quality of life, diabetes-related stress and treatment satisfaction are correlated with glycemia risk index (GRI), time in range and hypoglycemia/hyperglycemia components in type 1 diabetes
Source: Endocrine. 2024 May 24;86(1):186–93. doi: 10.1007/s12020-024-03846-9 (PMC11445287; doi:10.1007/s12020-024-03846-9)
Supplement: Supplementary file 1 — Supplementary tables [file 12020_2024_3846_MOESM1_ESM.docx]

**TABLE 1: CLINICAL, METABOLIC, GLUCOMETRIC AND PSYCHOSOCIAL FEATURES OF PATIENTS UNDER TREATMENT WITH CSII OR MDI**

| **PARAMETER** | **CSII** | **MDI** | **p-value** |
| --- | --- | --- | --- |
| **Number of patients** | 20 | 71 |  |
| **Mean age (years)** | 34,0 (10,8) | 36,9 (13,1) | NS |
| **Duration of diabetes (years)** | 20,6 (11,0) | 17,3 (11,2) | NS |
| **Mean HbA1C (%)**  **NFCC mmol/mol** | 7,0 (0,5)  53 (5,0) | 7,6 (1,1)  60,0 (12,0) | <0,01 |
| **Mean Glucose (mg/dl)** | 155,9 (18,5) | 175,9 (38,3) | <0,01 |
| **Nº daily scans** | 13,0 (8,6) | 8,9 (3,7) | NS |
| **% Sensor Use** | 93,0 (8,9) | 90,7 (11,0) | NS |
| **% TIR (70-180 mg/dL)** | 61,3 (10,2) | 51,7 (16,7) | <0,01 |
| **% TAR (>250 mg/dL)** | 9,1 (5,5) | 17,0 (13,7) | <0,01 |
| **%TAR (181-250 mg/dL)** | 22,9 (6,9) | 24,4 (8,4) | NS |
| **% TBR (54-69 mg/dL)** | 4,9 (2,4) | 4,2 (3,0) | NS |
| **% TBR (<54 mg/dL)** | 1,8 (2,2) | 2,6 (3,3) | NS |
| **SD (mg/dl)** | 61,2 (10,4) | 69,2 (22,6) | =0,027 |
| **CV (%)** | 39,3 (5,1) | 40,6 (7,8) | NS |
| **GMI (%)** | 7,1 (0,6) | 7,8 (1,3) | <0,01 |
| **GRI** | 50,0 (13,5) | 63,7 (23,4) | <0,01 |
| **CHypo** | 5,7 (3,8) | 5,9 (5,1) | NS |
| **CHyper** | 20,6 (8,3) | 29,2 (15,2) | <0,01 |
| **DQoL TOTAL** | 81,6 (23,5) | 87,0 (23,1) | NS |
| **DQoL Satisfaction** | 29,5 (10,4) | 32,7 (9,3) | NS |
| **DQoL Impact** | 31,4 (8,2) | 33,3 (9,8) | NS |
| **DQoL Social Concern** | 11,9 (5,1) | 12,4 (5,7) | NS |
| **DQoL Diabetes Concern** | 8,9 (2,9) | 9,9 (6,9) | NS |
| **DDS TOTAL** | 37,5 (20,1) | 46,9 (21,6) | NS |
| **DDS Emotional burden** | 13,8 (7,6) | 14,2 (6,0) | NS |
| **DDS Doctor-related distress** | 6,8 (5,4) | 9,8 (7,5) | =0,047 |
| **DDS Treatment-related distress** | 11,6 (6,0) | 15,2 (7,1) | =0,042 |
| **DDS Interpersonal-related distress** | 6,0 (4,4) | 7,1 (4,6) | NS |
| **DKQ2** | 26,6 (4,6) | 24,8 (4,8) | NS |
| **VAS** | 9,3 (1,2) | 8,7 (1,2) | NS |
| **Education level (%)** | Primary Education 10  Secondary Education 40  Higher Education 50 | Primary Education 15.5  Secondary Education 60.6  Higher Education 23.9 | P=0.057 |

T1D: Type 1 diabetes. CSII: Continuous Subcutaneous Insulin Infusion; MDI: Multiple Daily Insulin Injections; TIR: Time in range; TAR: Time above range; TBR: Time below range; CV: coefficient of glycemic variability; GMI: Glucose management indicator; SD= Standard desviation GRI: Glycemia Risk Index; CHypo: Hypoglycemia component; CHyper: Hyperglycemia component; DQoL: Diabetes Quality of Life; DQoL Satisfaction category; DQoL Impact category; DQoL Social concern category; DQoL Diabetes concern category; DDS: Diabetes Distress Scale; DDS Emotional burden category; DDS Doctor-related distress category; DDS Treatment-related distress category; DDS Interpersonal-related distress category; DKQ2: Diabetes Knowledge Questionnaire 2; VAS: Visual Analogue Scale; Clarke: Clarke’s Questionnaire; NS: not significant.

**TABLE 2: RELATIONSHIP BETWEEN THE SCORES ON QUALITY OF LIFE QUESTIONNAIRES AND THE VALUES OF CLARK TEST**

| PARAMETERS | Clarke Test  <3 | Clarke Test  >3 | p-value |
| --- | --- | --- | --- |
| DQoL | 84.0 (22.3) | 92.7 (26.1) | ns |
| DDS | 45.6 (21.0) | 43.9 (24.4) | ns |
| DKQ2 | 25.5 (4.9) | 23.9 (4.3) | ns |
| VSA | 8.9 (1.2) | 8.7 (1.4) | ns |

GRI: Glycemia Risk Index; DQoL: Diabetes Quality of Life; DDS: Diabetes Distress Scale; DKQ2: Diabetes Knowledge Questionnaire 2; VAS: Visual Analogic Scale.
